# P04 Surgical antibiotic prophylaxis adherence: before and after antimicrobial stewardship team impact in a south Indian secondary care hospital

**DOI:** 10.1093/jacamr/dlae136.008

**Published:** 2024-08-23

**Authors:** Suresh Kumar Dorairajan, Sowmiya Srinivasan, Safiya Banu Sadik Basha, Sanju Menaka Sivakumar, Saravanan Rajavel, Sakthi Atchitha Sudalai Kannan, Rithika Mari, Santhiya Kuppusamy, Monish Kumar Koteeswaran

**Affiliations:** Sundaram Medical Foundation, Chennai, India; Sundaram Medical Foundation, Chennai, India; Sundaram Medical Foundation, Chennai, India; Sundaram Medical Foundation, Chennai, India; Sundaram Medical Foundation, Chennai, India; Sundaram Medical Foundation, Chennai, India; Sundaram Medical Foundation, Chennai, India; Sundaram Medical Foundation, Chennai, India; Sundaram Medical Foundation, Chennai, India

## Abstract

**Background:**

Surgical antibiotic prophylaxis (SAP) is the major reason for antibiotic usage in secondary care hospitals. Implementing international guidelines, such as ASHP, is feasible in large tertiary care hospitals due to the presence of hospital antibiotic stewardship teams. However, it is challenging in resource-limited secondary care hospitals. The impact of antibiotic stewardship teams on SAP in secondary care hospitals is limited in the developing world.

**Objectives:**

To assess the impact of the hospital’s antibiotic stewardship team on the SAP adherence in a secondary care hospital in India.

**Methods:**

This prospective pre- and post-intervention study was conducted in a 200-bed South Indian secondary care hospital between May and October 2023. The hospital’s antimicrobial stewardship team (AMST), guided by an infectious disease physician, audited the case records of surgical patients, and adherence to SAP guidelines was recorded. In the pre-intervention phase (May to July 2023), no meetings occurred between the hospital surgical and antimicrobial stewardship teams, with only a few random SAP audits. In the post-intervention phase (August to October 2023), regular meetings facilitated systematic monitoring of all surgeries for SAP adherence, with data recorded in Google forms and the impact analysed using descriptive statistics.

**Results:**

A total of 1520 surgeries were conducted during the study period, with 767 in the pre-intervention period and 753 in the post-intervention period. In the pre-intervention period, 281 elective clean and clean-contaminated surgeries were audited, of which 200 received antibiotics. However, only 27% received appropriate antibiotics according to SAP guidelines, with a mean duration of SAP at 6.62 days. In the post-intervention period, all 753 surgeries were audited, and 350 elective clean and clean-contaminated surgeries received antibiotics. In this phase, 57.7% of the surgeries adhered to appropriate SAP, with a mean duration of SAP at 5.89 days (Table 1).

**Conclusions:**

AMST significantly improved SAP guidelines adherence, enhancing appropriate antibiotic use and reducing duration. Future efforts should focus on continuous monitoring and education for sustained improvement.

**Table 1.** Hospital AMST impact on SAP adherence

	Pre-intervention	Post-intervention
Total surgeries	1520	
	767	753
Audited	281	753
Antibiotics given	200	350
Appropriate choice, %	27	57.7
Mean duration, days	6.62	5.89

